# Cyanobacteria as Candidates to Support Mars Colonization: Growth and Biofertilization Potential Using Mars Regolith as a Resource

**DOI:** 10.3389/fmicb.2022.840098

**Published:** 2022-07-05

**Authors:** Inês P. E. Macário, Telma Veloso, Silja Frankenbach, João Serôdio, Helena Passos, Clara Sousa, Fernando J. M. Gonçalves, Sónia P. M. Ventura, Joana L. Pereira

**Affiliations:** ^1^Department of Biology, Centre for Environmental and Marine Studies, University of Aveiro, Aveiro, Portugal; ^2^CICECO – Aveiro Institute of Materials, Department of Chemistry, University of Aveiro, Aveiro, Portugal; ^3^Laboratório Associado, Centro de Biotecnologia e Química Fina, Escola Superior de Biotecnologia, Universidade Católica Portuguesa, Porto, Portugal

**Keywords:** cyanobacteria, microalgae, MGS-1 regolith simulant, *Lemna minor*, biofertilization

## Abstract

Cyanobacteria are indicated as organisms that can possibly support Mars colonization, contributing to the production of oxygen and other commodities therein. In this general context, the aim of this work was to evaluate the ability of three species of cyanobacteria (*Anabaena cylindrica, Nostoc muscorum*, and *Arthrospira platensis*) and a green microalga (*Chlorella vulgaris*) to grow using only the resources existing in Mars, i.e., water and Martian regolith stimulant (MGS-1), under an Earth-like atmosphere. A Martian regolith extract was produced and used as a culture medium to grow these species. Their growth was assessed during a period of 25 days, using optical density and fluorometric parameters. After this period, the possible contribution of end-of-life cyanobacteria/microalga as biofertilizing agents was also assessed, using the macrophyte *Lemna minor* as a vegetable model. Among the three species, *N. muscorum* showed the best growth performance when compared to the other species, while *A. platensis* and *C. vulgaris* were not able to thrive on Mars regolith extract. Therefore, *N. muscorum* should be the target of future studies not only due to their role in oxygen production but also due to their possible use as a food source, as many members of the *Nostoc* genus. Cyanobacteria and microalgae (*A. platensis* and *C. vulgaris*) showed good abilities as biofertilizing agents, i.e., they stimulated biomass (i.e., dry weight) production at levels comparable to the plants that grew on standard synthetic medium. The highest yield was reached with *A. platensis*, while the lowest was achieved using the media with *N. muscorum.* FTIR-ATR (Fourier transform infrared with attenuated total reflectance) spectroscopy showed that the differences between the plants grown on media with or without Martian regolith seem to be related mainly to polysaccharides.

## Introduction

Every day, the dream to colonize Mars is closer to becoming a reality. However, the challenges for human survival on Mars are huge, and one of the key issues is ensuring low-to-no dependence on Earth’s resources. Therefore, several bioregenerative life-support systems for recycling food, water, and gasses are under development (e.g., Nelson et al., 2010; Häder, 2020), and one pursued solution is the use of Martian resources to support life. Indeed, Mars can possibly provide water, solar energy, carbon, nitrogen, and other nutrients (Cockell, 2014).

In this context, cyanobacteria are pointed out as potentially suitable organisms for use in basic life-support systems (Arai et al., 2008; Arai, 2009; Kimura et al., 2015; Verseux et al., 2016, 2021). This is because cyanobacteria are simple (i.e., prokaryotic) organisms, easy to culture with low nutritional requirements, and able to live in a wide range of conditions and ecosystems (e.g., from freshwater to salt and brackish waters, rainforests, deserts and other terrestrial habitats, and even in the air), including under extreme environmental conditions (Gaysina et al., 2018). Furthermore, cyanobacteria are oxygenic photosynthetic bacteria and hence produce oxygen, which is a remarkably limited resource on Mars. Indeed, this critical element to support human survival only represents 0.13% of the Mars atmosphere, while it represents 21% of the Earth’s atmosphere (Verseux et al., 2016). On Mars, oxygen is expected to be produced on-site by photosynthetic organisms and/or through physicochemical methods, such as the electrolysis of the regolith brine (Gayen et al., 2020).

Compared to plants, photosynthetic microorganisms are better at capturing solar energy on a volume-to-output basis and therefore are very efficient oxygen producers. For example, an industrially cultivated *Arthrospira* strain has been found to produce ca. 16.8 tons of O_2_ ha^–1^ year^–1^, which suggests a much higher O_2_ production rate per unit area than trees that have been shown to release ca. 2.5–11 tons of O_2_ ha^–1^ year^–1^ (Verseux et al., 2016). The production of oxygen by cyanobacteria is so impressive that estimates pointed out that the marine cyanobacterium *Prochlorococcus* alone can be responsible for ca. 5% of global photosynthesis (Pennisi, 2017). Moreover, plants are more demanding with regard to environmental tolerance ranges and require more space and resources; thus, they are more costly than cyanobacteria, particularly when Mars culturing is equated. Besides oxygen, these organisms are able to produce a wide range of other interesting compounds with several applications, e.g., amino acids, pigments, lipids, polysaccharides, and proteins, with applications in the food and pharmaceutical industries (Eriksen, 2008; Sekar and Chandramohan, 2008; Macário et al., 2021). Different species can also be used directly as food sources (e.g., *Arthrospira* and *Nostoc* sp.; Gao, 1998; Johnson et al., 2008), to produce biofuels (Raheem et al., 2018), in bioremediation (Fawzy and Mohamed, 2017), and as biofertilizers (Grzesik and Kalaji, 2017). These features render cyanobacteria highly promising for culturing and exploitation in harsh environments under resource scarcity conditions, such as on Mars.

Indeed, cyanobacteria (e.g., *Chroococcidiopsis* sp.) were already proven to be able to survive in space, i.e., on the outer surface of the International Space Station at extreme temperature fluctuation, vacuum conditions, and high UV and cosmic radiation (de Vera et al., 2019). In addition, besides the production of oxygen, their biomass can be used to supply different commodities, thus contributing to the development of sustainable and less Earth-dependent life-support systems. Therefore, the primary objective of the present study was to evaluate the ability of several species of cyanobacteria (*Anabaena cylindrica, Nostoc muscorum*, and *Arthrospira platensis*) to grow using only the resources existing on Mars (i.e., water and Martian regolith). For performance comparison with the cyanobacteria, we also tested the green microalga *Chlorella vulgaris*, an eukaryote that can be also studied for most of the previously mentioned applications (Mostafa, 2012). In this work, we also addressed the suitability of end-of-life cyanobacteria/microalgae as biofertilizing agents for vegetable cultivation, considering that plants are an essential component of long-term, bioregenerative life-support systems in Mars (Heinse et al., 2007). For this purpose, we tested cyanobacteria/microalgae cultures grown on Mars regolith extract as a biofertilizer using the macrophyte *Lemna minor* as a preliminary model.

## Materials and Methods

### Cyanobacteria and Microalgae Cultures and Culturing Conditions

Three species of filamentous cyanobacteria (*Anabaena cylindrica* PCC 7122, *Nostoc muscorum* UTAD_N213, and *Arthrospira platensis* UTEX LB 2340) and one species of microalgae (*Chlorella vulgaris*) were used. These species were chosen due to their potential edibility (species from the genus *Nostoc*, *Arthrospira*, and *Chlorella* are currently considered edible food resources in many countries; García et al., 2017), or high nutritional value, e.g., *Anabaena*, which is an easily cultivable genus bearing high nutritional value (Rosales-Loaiza et al., 2017).

Cultures are established for many years in our laboratory in Woods Hole MBL synthetic medium (Nichols, 1973) or in Spirulina medium (for the culturing of *A. platensis*). All species were progressively acclimated for more than 6 months to a minimum nutrient supply (17% of synthetic media and 83% of ultrapure water, determined in preliminary trials as the maximal dilution supporting cell growth within the defined experimental period). The cultures were kept in 100 mL Erlenmeyer vessels with 50 mL of medium in an incubation chamber at 20 ± 2°C with 16-h light:8-h dark photoperiod (7 μmol of photons m^–2^ s^–1^; Quantum meter MQ-200, Apogee Instruments, Logan, UT, United States) provided by cool white fluorescent tubes.

### Mars Regolith Extract Preparation and Characterization

Mars Global Simulant (MGS-1) was obtained from the Center of Lunar and Asteroid Surface Science (Orlando, FL, United States). This simulant was used to prepare an aqueous extract serving as the growth medium for cyanobacteria and microalgae (see section “Cyanobacteria and Algae Growth Experiment”). Suspensions of the regolith in ultrapure water were prepared following a 4:1 (v:w) ratio, and then autoclaved at 121°C for 1 h. The autoclaved suspension was left for the settling of particulate material at room temperature, and then centrifuged (4,111 × *g*, 14°C, 5 min) for the final collection of the overlying fraction. This aqueous extract was autoclaved again (121°C, 30 min) to ensure sterilization.

The composition of the extract was determined by total X-ray fluorescence (TXRF) using a benchtop Picofox S2 (Bruker Nano) spectrometer with a molybdenum X-ray source. Both quartz glass and acrylic sample carriers were used. Sample carriers were pretreated with 10 μL of a solution of silicon in isopropanol and dried in a heat plate at 80°C. Then, 10 μL of yttrium internal standard solution was added to 1 g of aqueous extract sample. A total of 10 μL of this mixture was added to treated sample carriers, which were dried under vacuum for 1 h and analyzed in the TXRF spectrometer for 300 s.

### Cyanobacteria and Microalgae Growth Experiment

The four test species were cultured for 25 days in triplicate, under three treatments: ultrapure water, Mars regolith extract, and 17% MBL/Spirulina medium (i.e., *A. platensis* was cultured in 17% of Spirulina medium, while the remaining species were cultured in 17% of MBL medium). The species were cultured in 100 mL Erlenmeyer vessels with 75 mL of the respective treatment plus 5 mL of a 1-month-old inoculum, under incubation conditions as described for bulk cultures (section “Cyanobacteria and Microalgae Cultures and Culturing Conditions”). Two times a week, 2-mL samples were collected in a sterilized environment for the measurement of optical density and fluorometric parameters (see section “Spectroscopic and Fluorometric Measurements”).

### *Lemna minor* Growth Experiment

*Lemna minor* was used as a vegetable model to address the biofertilizer potential of cyanobacterial cultures because of its small size, low requirements for culturing in terms of space and volume of culture media, as well as considering the availability of standard guidelines to accurately evaluate growth performance. Following the growth experiment (section “Cyanobacteria and Microalgae Growth Experiment”), the cultures of cyanobacteria/microalgae that grew in Mars regolith extract were tested as a culturing medium for the macrophyte *L. minor* (see section “Analysis of the Media Used for the *Lemna minor* Growth Experiment” for the brief characterization of these media). Five culture media for *L. minor* were compared in this test: (i) Steinberg medium (standard medium for *Lemna* sp. growth; OECD, 2006), (ii) ultrapure water, (iii) Mars regolith extract prepared as detailed in Section “Mars Regolith Extract Preparation and Characterization,” (iv) the media of 25-day-old (whole) cultures of cyanobacteria/microalgae in Mars regolith extract after filtering through 0.45-μm mesh size cellulose acetate membranes, and (v) 25-day-old (whole) cultures of cyanobacteria/microalgae in Mars regolith extract after sonication at 17 W (Vibra Cell, Sonics) until homogenization. These last two treatments (iv and v) addressed two hypotheses: metabolites released by cyanobacteria/microalgae into the extracellular environment are responsible for biofertilization capacities (treatment iv) and the biofertilization abilities are rather the result of the availability of the intracellular contents in the media (treatment v).

The growth assay with *L. minor* was performed following the appropriate OECD guidelines (OECD, 2006) adapted to the use of six-well plates (Kaza et al., 2007). Each treatment was tested in triplicate in wells filled with 10 mL of the test solution. The test was initiated by adding three macrophyte colonies of three fronds each, harvested from a weekly renewed laboratory culture in Steinberg medium held at 23 ± 2°C under continuous illumination (ca. 74 μmol of photons m^–2^ s^–1^; Quantum meter MQ-200, Apogee Instruments, Logan, UT, United States) (OECD, 2006). The test plates were incubated for 7 days under the same culturing conditions as used for the *L. minor* culture. Fluorometric parameters F_*v*_/F_*m*_ were analyzed on days 0, 2, 4, and 7, according to the methodology described in Section “Spectroscopic and Fluorometric Measurements.” At the end of the test, the fronds were counted and oven-dried (at least 24 h at 60°C) for dry weight measurements. The effects of the treatments were assessed regarding the yields considering the frond number and dry weight (the initial dry weight was measured in eight additional groups composed of three colonies with three fronds each) records. The dried fronds were then used for the FTIR-ATR (Fourier transform infrared with attenuated total reflectance) analysis (Section “Infrared Spectra Acquisition”).

### Spectroscopic and Fluorometric Measurements

The growth of cyanobacteria/microalgae cultures was monitored two times a week by measuring the optical density (OD) records at 440 nm (Shimadzu UV 1800, Shimadzu Corporation, Kyoto, Japan). Furthermore, an imaging chlorophyll fluorometer (Open FluorCAM 800-O/1010, Photon Systems Instruments; Brno, Czech Republic) was used to capture the maximum photosynthetic quantum yield of Photosystem II (F_*v*_/F_*m*_) after 15 min of dark adaptation. The excitation light peaks at 621 nm with a 40 nm bandwidth with a saturating pulse intensity of about 7,000 μmol quanta m^–2^ s^–1^ and the duration set to 0.8 s. The fluorescence signal emitted by chlorophyll *a* was captured by using a 2/300 CCD camera (CCD381) with an F1.2 (2.8–6 mm) objective, which resulted in images with 512 × 512 pixels and a spectral range of 695–780 nm. Images were processed using the FluorCam7 software (Photon Systems Instruments; Brno, Czech Republic). Fluorometric measurements were made on each culture of cyanobacteria/microalgae, in triplicate. For this purpose, aliquots of 2 mL of each culture were transferred into six-well plates. In the case of *L. minor*, the measurements were made directly in the six-well plates of the test. The maximum quantum yield of PS II, or maximal PS II efficiency (F_*v*_/F_*m*_), was calculated according to eq. 1 (Ogawa et al., 2017).


(1) $$\frac{F_{v}}{F_{m}}=\frac{F_{m}-F_{o}}{F_{m}}$$

where F_*o*_ is the minimum fluorescence in dark-adapted samples, and F_*m*_ is the maximal fluorescence after exposure to a saturating light pulse.

### Infrared Spectra Acquisition

The mid-infrared spectra of lyophilized and ground *Lemna* plants were obtained on a Fourier transform PerkinElmer Spectrum BX FTIR System spectrophotometer (United States) with a DTGS detector. Spectra were acquired in diffuse reflectance mode through a PIKE Technologies Gladi attenuated total reflectance (ATR) accessory within the wavenumber interval of 4,000 to 600 cm^–1^, with a resolution of 4 cm^–1^. Each spectrum resulted from 32 scan co-additions. For each sample, a small portion was transferred to the ATR crystal and constant pressure was applied. The ATR crystal was cleaned and the background was acquired between each sample. For each *L. minor* replicate, three spectra were acquired (instrumental replicates).

### Analysis of the Media Used for the *Lemna minor* Growth Experiment

The samples of the media used in the *L. minor* growth experiment bearing cyanobacteria/microalgae were analyzed by TXFR, following the procedure described in section “Mars Regolith Extract Preparation and Characterization,” for several elements. Nitrates and orthophosphate were also quantified by colorimetric methods (Aqualytic^®^ Kits; Tests no. 265 and no. 320).

### Data Analysis

Data regarding the monitoring of the culture growth (optical density and F_*v*_/F_*m*_) were graphically expressed as the mean ± standard deviation (SD) of three replicates. The effect of the different media, composed of different cyanobacteria/microalgae cultures (see section “*Lemna minor* Growth Experiment”), on *L. minor* yield was statistically addressed using a one-way ANOVA approach, followed by the post-hoc Tukey’s test to distinguish differences among the groups. An alpha level of 0.05 was considered in these analyses. FTIR-ATR spectra were processed with standard normal variate (SNV) (Næs et al., 2002), followed by the application of a Savitzky–Golay filter (15 smoothing points, 2^nd^ order polynomial, and first derivative) (Savitzky and Golay, 1964). Spectra were additionally mean-centered and analyzed by principal component analysis (PCA) (Jolliffe, 1986). All chemometric models were performed in Matlab version 9.5 Release 2018b (MathWorks) and PLS Toolbox version 8.7 (2019) for Matlab (Eigenvector Research, Manson, WA).

## Results

### Analysis of Mars Regolith Extract

The composition of the Mars regolith extract was similar to that provided by the manufacturer (Supplementary Table 1). TXRF analysis revealed that the Mars regolith extract was rich in S, K, and Ca and contained smaller concentrations of Mn, Fe, and Sr, and trace concentrations of Cu, Zn, Br, and Rb (Table 1).

**TABLE 1:** Elements identified in the Mars regolith extract by total X-ray fluorescence (TXRF).

Element	[M]/(mg L^–1^)	±σ/(mg L^–1^)
S	338	2
K	5.4	0.1
Ca	90.7	0.4
Mn	0.228	0.007
Fe	0.235	0.006
Cu	0.009	0.002
Zn	0.030	0.002
Br	0.025	0.001
Rb	0.025	0.001
Sr	0.247	0.003

*In the blank (ultrapure water), only S, K, and Ca were detected at trace levels (ppm).*

### Cyanobacteria and Microalgae Growth Experiment

**FIGURE 1:**
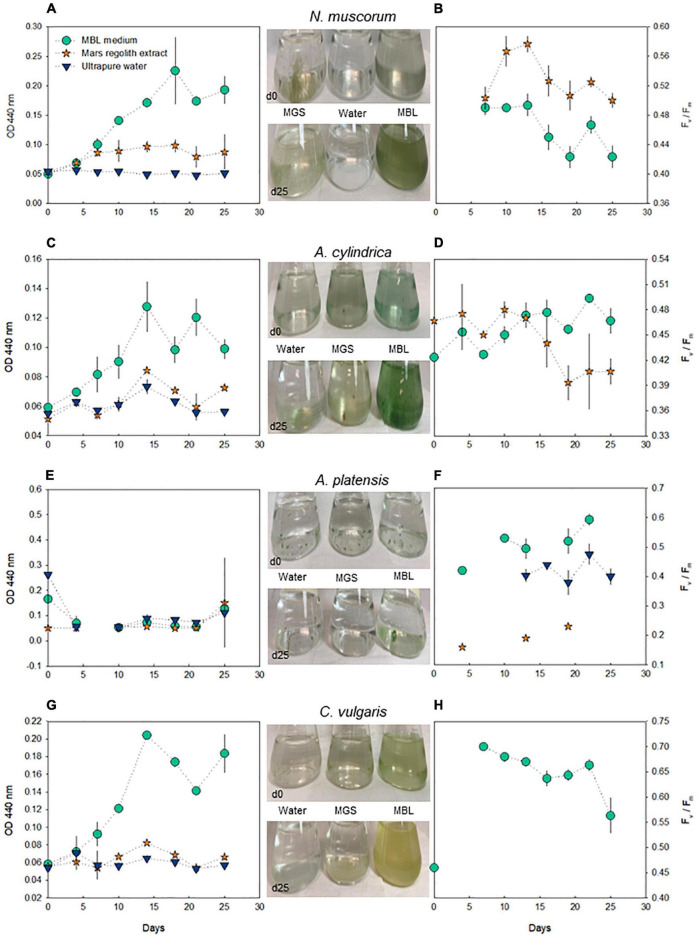
Profiling of *N. muscorum*, *A. cylindrica*, *A. platensis*, and *C. vulgaris* throughout a monitoring period of 25 days, regarding OD measurements at 440 nm **(A,C,E,G)** and PS II efficiency (F_*v*_/F_*m*_) **(B,D,F,H)** (only data with F_0_ > 100 are depicted). Marks represent the mean values and the error bars represent the standard deviation (*n* = 3). In the central panel, illustrative photos are provided (full documentation in Supplementary Figure 1).

The growth of cyanobacteria and microalgae was monitored for 25 days using optical density measurements. Additionally, fluorometry was used to assess PS II efficiency (F_*v*_/F_*m*_). The highest optical density was recorded when the species were cultured in diluted synthetic media, while a lower growth was observed when the species were cultured in the treatment with ultrapure water (Figure 1). *N. muscorum* and *A. cylindrica* were able to grow in Mars regolith extract, although with lower biomass yield compared to the growth in MBL medium. However, *A. platensis* showed only mild growth in this treatment.

*N. muscorum* exhibited the most consistent growth, as well as the highest OD records, both in the diluted synthetic medium and in Mars regolith extract (Figure 1, left-hand panel). Regarding the maximal PS II efficiency (F_*v*_/F_*m*_), *N. muscorum* always recorded values above 0.40 in these two treatments, although with a decreasing tendency through time (Figure 1B). Interestingly, *N. muscorum* showed higher values of F_*v*_/F_*m*_ in Mars regolith extract than in MBL. *A. cylindrica* recorded stable F_*v*_/F_*m*_ values when grown in Mars regolith extract, but the photosynthetic efficiency remarkably decreased in cultures grown in the regolith extract from day 15 onward (Figure 1D). Indeed, after the 3^rd^ week of culturing in the regolith extract, the biomass of *A. cylindrica* became brownish, which was reflected in a decrease in F_*v*_/F_*m*_ values (Supplementary Figure 1). The growth of both *N. muscorum* and *A. cylindrica* was so limited in ultrapure water that the fluorescence signal of the samples was not detected, preventing F_*v*_/F_*m*_ measurements. Conversely, *A. platensis* growing in ultrapure water showed F_*v*_/F_*m*_ values higher than in the regolith extract (Figure 1F). However, this outcome should be carefully interpreted as the colonies of this species clogged strongly (Supplementary Figure 1), preventing reliable measurements of optical density primarily and also fluorescence parameters to some extent. Despite this constraint, the direct observation of these cultures allowed for the confirmation of mild growth, specifically in diluted Spirulina medium. In the case of the microalga *C. vulgaris*, only the culture in diluted MBL produced sufficient biomass to support fluorescence measurements, which showed a decrease in the last days (Figure 1H).

### *Lemna minor* Growth Experiment

**FIGURE 2:**
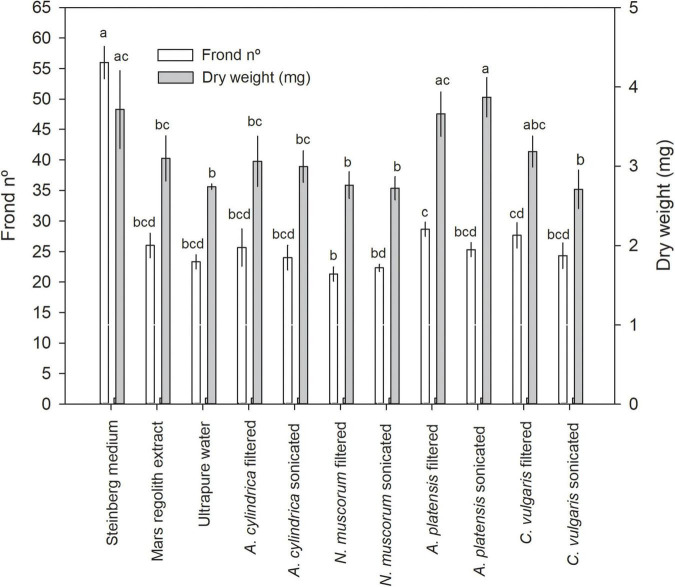
*Lemna minor* biomass yield (in frond number or dry weight) after 7 days of culturing in Steinberg medium, Mars regolith extract, ultrapure water, and cyanobacteria/microalgae grown in Mars regolith extract, either filtered or sonicated. Bars represent the mean of three replicates and the error bars represent the standard deviation. Different letters indicate significant differences among treatments (Tukey’s test; *p* < 0.05).

*Lemna minor* was used herein to test for the potential of cyanobacteria and microalgae cultures as biofertilizing agents (see photographic documentation in Supplementary Figure 2). As expected, *L. minor* tested in Steinberg medium recorded the best yields with regard to both the number of fronds and dry weight (Figure 2). They grew better in Mars regolith extract than in ultrapure water, although the difference was not statistically significant. *L. minor* grown in cyanobacteria/microalgae cultures showed frond number yields close to those obtained with regolith extract or ultrapure water, with the exception of *A. platensis* (filtered and sonicated) and *C. vulgaris* (filtered) that showed a dry weight statistically similar to the controls with Steinberg medium (Figure 2) (*F*_10,32_ = 79.190 with *p* < 0.001 for dry weight; *F*_10,32_ = 8.040 with *p* < 0.001 for frond number). Among *L. minor* cultures in cyanobacteria/microalga, the highest biomass was reached with *L. minor* grown in filtered *A. platensis* medium (28.7 fronds) and an average dry weight yield of 3.66 mg, which did not significantly differ from the dry weight reached by plants cultured in Steinberg medium (3.71 mg), as evidenced in Figure 2. An even higher dry weight yield was reached with sonicated *A. platensis* (3.87 mg), which also did not significantly differ from the records for the Steinberg control. The lowest *L. minor* growth was recorded with *N. muscorum* (21.3 fronds and 2.72 mg of dry weight). No significant differences were found between filtered and sonicated cultures of the same species in promoting the growth of *L. minor.*

**FIGURE 3:**
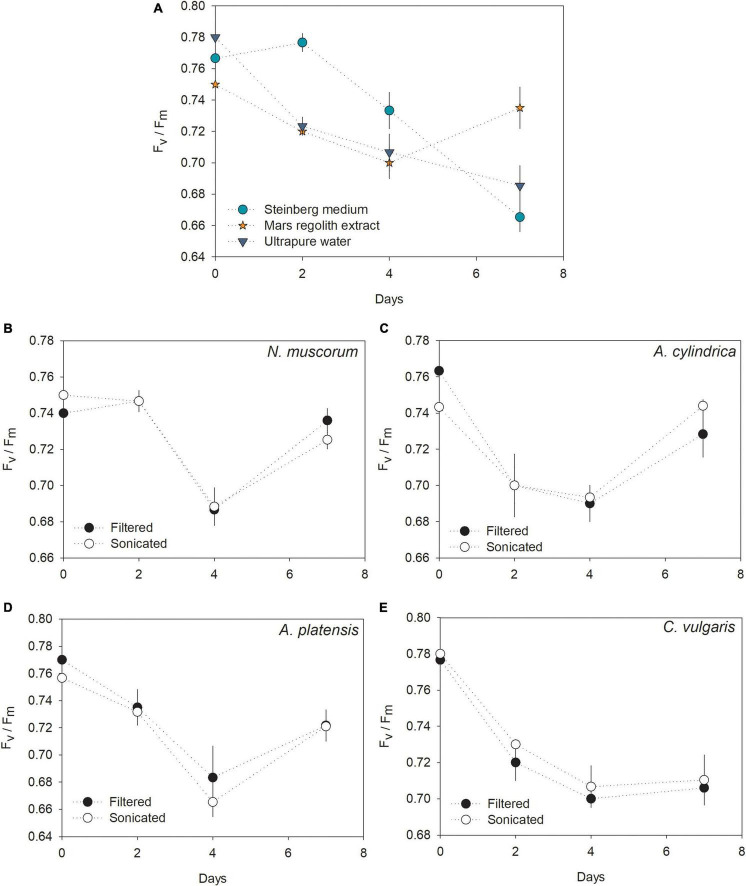
**(A–E)** PS II efficiency (F_*v*_/F_*m*_) over time for *L. minor* cultured in Steinberg medium, Mars regolith extract, ultrapure water, and cyanobacteria/microalgae cultured in Mars regolith extract after filtration or sonication. Marks represent the mean values and the error bars represent the standard deviation (*n* = 3).

The highest PS II yield was recorded for *L. minor* growing in Steinberg medium, showing a decrease after 2 days. A decreasing pattern was also recorded for plants growing in ultrapure water (Figure 3A). Interestingly, F_*v*_/F_*m*_ values recorded for *L. minor* growing in the regolith extract decreased along the first 4 days and then increased, almost reaching initial levels by day 7. There was no apparent difference in the F_*v*_/F_*m*_ profiles of *L. minor* growing in filtered or sonicated cultures of the same cyanobacteria/microalgae species (Figure 3). *L. minor* grown in the media with *N. muscorum*, *A. cylindrica*, and *A. platensis* recorded F_*v*_/F_*m*_ profiles similar to plants grown in regolith extract only, with a decrease in F_*v*_/F_*m*_ values until day 4, followed by a recovery to values closer to the initial levels by day 7 (i.e., F_*v*_/F_*m*_ (0.72 – 0.74)), higher than that recorded for plants grown in Steinberg medium (0.66) and ultrapure water (0.68). Differently, *L. minor* grown in the medium with *C. vulgaris* showed a decrease in F_*v*_/F_*m*_ values until day 4, but from then onward the records stabilized.

**FIGURE 4:**
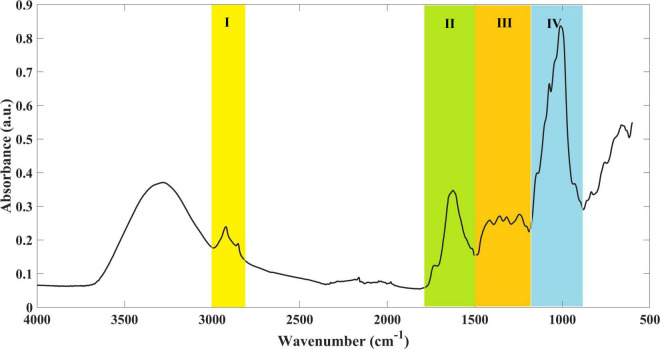
Example of an infrared spectrum of *L. minor*. Four major regions (I–IV) were identified, representing major classes of compounds.

The analysis of the nutritional composition of *L. minor* grown in different treatments was assessed by FTIR-ATR, in order to understand if profiles were different depending on the treatment. The infrared spectrum of *L. minor* presented typical bands, which can be attributed to some major classes of compounds (Figure 4). Region I (between 3,000 and 2,800 cm^–1^) is dominated by –CH_3_ and –CH_2_ stretching vibrations. Region II (between 1,800 and 1,500 cm^–1^) is characterized by the C=O stretching vibration at 1,738 cm^–1^, indicating the presence of ester-containing compounds from the cell wall and membrane lipids at 1,656 and 1,563 cm^–1^ vibrations of amide I and amide II, respectively, and at 1,513 cm^–1^ vibrations of the aromatic ring-like lignin derivatives. In region III (between 1,500 and1,200 cm^–1^), it is possible to identify –CH_3_ and –CH_2_ bending (1,460 and 1,400 cm^–1^). Finally, in region IV (between 1,200 and 900 cm^–1^), the intense infrared absorption indicates a high content of polysaccharides (C–H bending and/or C–O and C–C stretching of cellulose and hemicellulose).

**FIGURE 5:**
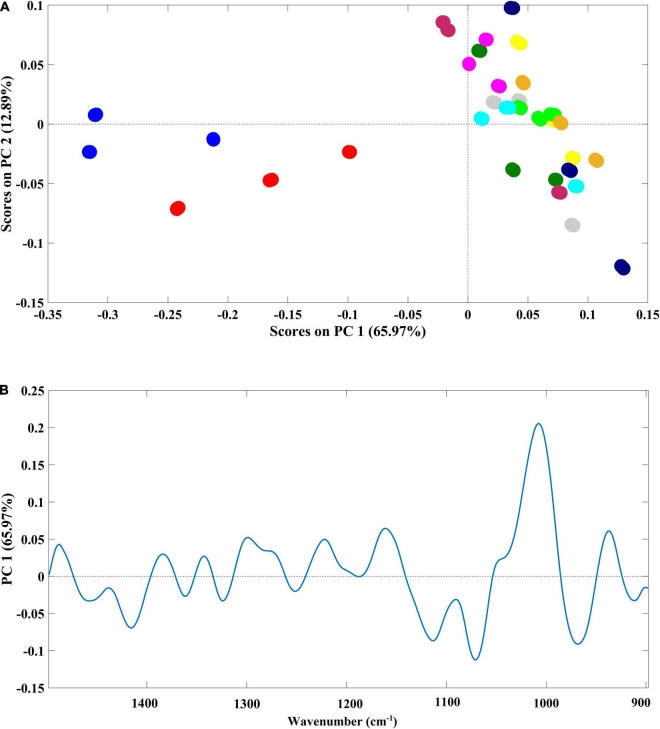
**(A)** Principal component analysis (PCA) scatter plot based on PC1 and PC2 scores (better representing the separation between sample groups); and **(B)** loading plot of the PCA model, evidencing changes in the chemical composition of *L. minor* after the growth period in 11 different media. Samples were color-coded by growth medium: 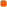 ultrapure water; 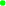 Mars regolith extract; 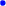 Steinberg medium; 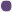 *A. cylindrica* filtered; 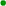 *A. cylindrica* sonicated; 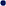 *N. muscorum* filtered; 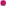 *N. muscorum* sonicated; 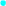 *A. platensis* filtered; 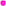 *A. platensis* sonicated; 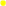 *C. vulgaris* filtered; 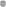 *C. vulgaris* sonicated.

A high spectral similarity was found among the samples. However, two main groups were clearly identified and separated through the PC1, which contributes to around 66% of the spectral variability (Figure 5A). One cluster (negative part of PC1) was composed of samples grown in ultrapure water and in Steinberg medium, while the second cluster (positive part of PC1) was composed of samples grown in Mars regolith extract and cyanobacteria/microalgae cultured in Mars regolith extract. Within this second group, there was no resolution in the analysis to discriminate among the different cyanobacteria/microalgae species. Indeed, the infrared spectra of this group were randomly widespread across the second PC (PC2) (Figure 5A). The PCA model loadings on the PC1 (where the discrimination between the presence/absence of Mars soil simulant occurs) (Figure 5B) denote that the spectral region mainly accounted for the referred discrimination in the region between 1,200 and 900 cm^–1^ (with the higher loading values). It is known that this spectral region is dominated by polysaccharide vibrations (Wilson et al., 2000), indicating that this class of compounds is the one that mostly differs in the *Lemna* plants grown in the presence or absence of Mars regolith extract.

Analysis of the composition of the media with cyanobacteria/microalgae used in the *L. minor* growth experiment denoted the presence of sulfur, calcium, potassium, manganese, nitrates, and orthophosphates (Supplementary Figure 3 and Supplementary Table 2). The concentrations of sulfur and calcium in the media with cyanobacteria/microalgae were much higher than in the Mars regolith extract. However, between the supplemented species, the concentrations of these elements were similar. Potassium concentration was also higher in the media with cyanobacteria/microalgae and was particularly high in the treatment with *A. platensis*. Regarding manganese concentrations, species-supplemented treatments were similar to Mars regolith extract, except for filtered *N. muscorum* and *A. platensis*, which showed significantly lower records. Nitrates were detected only in *A. platensis* samples, while orthophosphate was found mostly in *A. platensis* and *N. muscorum* samples, with the highest concentration found for a sonicated *N. muscorum* sample.

## Discussion

The forecasts highlight the decade of the 2030s as the year of the launch of a human space flight mission to Mars (United States Congress, 2017). However, Martian conditions challenge human survival, and cyanobacteria can thrive using Martian resources (under an Earth-like atmosphere), supporting Mars colonization by contributing to oxygen production, as well as by becoming a feedstock for other applications (e.g., food supplements, biofertilizers, and biofuel production). Several works already proved that these organisms are able to survive by utilizing the resources available on Mars, namely, water and Martian regolith (Arai et al., 2008; Arai, 2009; Kimura et al., 2015; Verseux et al., 2021). On Mars, water can be found in the form of ice in the northern polar region (Shotwell, 2005) and in regolithic brines (Martínez and Renno, 2013; Lauro et al., 2021). Therefore, water can be “mined” and extracted for exploitation (e.g., Hoffman et al., 2016; Lordos et al., 2021). While having water is the first step for survival, the following could be the use of Martian soil. The composition of Mars soil is known, and simulants of Martian regolith are available for experiments (e.g., MGS-1).

Herein, the two components (i.e., water and Mars regolith) plus atmospheric CO_2_ and nitrogen that are also available on Mars (1.9% of N_2_ and 98% CO_2_;^1^ assessed on May 2022) were proven to be enough to support the survival and growth of cyanobacteria, on the basis of optical density and the fluorimetric parameter F_*v*_/F_*m*_. However, not all the tested species were able to grow satisfactorily in Mars regolith extract. *A. platensis* did not show quantifiable growth even in a synthetic medium, although the data from F_*v*_/F_*m*_ showed that the cells were alive and photographic documentation (Supplementary Figure 1) showed low but visible growth. The poor nutrient supply in the treatments supports this limited growth outcome. *C. vulgaris* was unable to grow, while *N. muscorum* and *A. cylindrica* thrived in Mars regolith extract. Verseux et al. (2021) also proved that the cyanobacterium *Anabaena* sp. was able to grow in MGS-1. Indeed, in the present study, the values of F_*v*_/F_*m*_ for *N. muscorum* were higher in Mars regolith extract than in synthetic medium, suggesting a better photosynthetic activity in this treatment. In this way, considering the main perspective of using cyanobacteria for oxygen production on Mars, *N. muscorum* can be highlighted among the tested species as a stronger candidate for further studies. Moreover, the use of the same biomass to produce multiple commodities is ideal in the context of low resource availability. Indeed, several species of *Nostoc* (e.g., *N. flagelliforme* and *N. commune*) are considered edible and have been used as food items for a long time in China, other Asian countries, and South America (Gao, 1998; Johnson et al., 2008). Studies on the nutritional profile of *N. muscorum* would be very important to evaluate if this species could be considered as a food resource for Martian crews.

Further studies are fundamental to understanding the suitability of the species with regard to other critical factors limiting their use on Mars, for instance, their ability to survive the journey and hold very low pressures and vacuum conditions (Arai et al., 2008) and their capacity to thrive under different atmospheres and lower pressures (see the studies of Verseux et al. (2021) with the cyanobacterium *Anabaena* sp. PCC 7938). It is worth mentioning in this context that specific devices are being developed to create a controlled and Earth-like environment, e.g., the A’MED (Arai’s Mars Eco-systems Dome), which is a closed dome where temperature, humidity, and light can be controlled, and protection is ensured against ultraviolet radiation, cosmic rays, and low pressures (Arai et al., 2008). This is an essential step for the culturing of these organisms, since in an unprotected Mars-like atmosphere, cyanobacteria would not likely thrive due to low pressure, absence of stable liquid water, and low nitrogen levels for diazotrophic growth (Verseux et al., 2021). Also, although water could be “mined,” this would require considerable effort and specific technology (e.g., Hoffman et al., 2016; Lordos et al., 2021). Therefore, it would be relevant to also evaluate the growth performance of cyanobacteria in a solid medium supplemented with MGS-1 (similar to what was made by Ye et al., 2021) in the near future.

Concerning the perspective of maximizing the use of Martian resources, only organisms with low nutritional requirements should be considered, given the poor composition of the Mars regolith. Indeed, the microalga *C. vulgaris* and the cyanobacterium *A. platensis* did not grow expressively in Mars regolith extract, suggesting that nutritional requirements for these organisms are higher than for the other two diazotrophic cyanobacteria (e.g., *N. muscorum* and *A. cylindrica*). Another possible explanation could be that these species have naturally slower growth dynamics or a higher lag phase, which would require an extended period of time to better observe changes.

Our results show that despite long-term survival and growth being unknown, poor nutrition provided by Mars regolith extract supports cyanobacterial growth in the short term (i.e., 1 month). Carbon, nitrogen, phosphorus, sulfur, potassium, and iron are the critical nutrients for cyanobacteria growth (Markou et al., 2014). Carbon can be obtained by cyanobacteria autonomously from atmospheric CO_2,_ and nitrogen can be obtained by some species from atmospheric nitrogen fixation (Markou et al., 2014). Diazotrophic species, such as *A. cylindrica* and *N. muscorum*, are able to fix atmospheric nitrogen through specialized structures (Issa et al., 2014), while other cyanobacterium species may also fix nitrogen through different mechanisms (Flores et al., 2015). On the other hand, *A. platensis* is not a nitrogen-fixing organism (Flores et al., 2015), which can explain the lack of growth in Mars regolith extract, similar to *C. vulgaris*. No traces of phosphorus were found in the Mars regolith extract analysis, but both cyanobacteria and microalgae can accumulate phosphorus reserves as polyphosphate granules, and these reserves can be used in cases of phosphorus shortage (Markou et al., 2014). As the cultures were established from laboratory stocks maintained under sub-optimal growth conditions, the mobilization of reserves should have been the mechanism supporting the appropriate phosphorous supply for cyanobacteria growing in Mars regolith extract.

Although phosphorus was not present in the tested Mars regolith extract, other important elements, such as potassium, manganese, calcium, and sulfur, were available. Potassium is, along with nitrogen and phosphorus, the main macronutrient for the growth of photosynthetic organisms (Markou et al., 2014). Cyanobacteria thylakoids bear ion channels where potassium flows to allow a maximal efficiency of photosynthesis (Checchetto et al., 2012). Potassium also plays a significant role as an activator of several enzymes involved in photosynthesis and respiration, protein and carbohydrate synthesis, and the regulation of the osmotic potential inside the cells (Markou et al., 2014).

While manganese is required as a micronutrient, it can be toxic in high (micromolar range) concentrations to cyanobacteria, thus negatively affecting photosynthesis, growth rates, and nitrogen fixation (Rueter and Petersen, 1987). Manganese is more toxic to cyanobacteria than to microalgae (Rueter and Petersen, 1987), thus it is unlikely that this element can explain the limited growth of *C. vulgaris* in Mars regolith extract. Calcium has a central role as a secondary messenger and signaling molecule in all living organisms (Clapham, 2007). It is also involved in atmospheric nitrogen fixation (Allen and Arnon, 1955), and its concentration has an influence on biomass and protein building (Walter et al., 2016). Sulfur is important for protein synthesis in general and in the biosynthesis of coenzymes (Schmidt, 1988), but contrarily to animals, for example, cyanobacteria are not dependent on the external supply of reduced sulfur compounds, since they can autonomously reduce sulfate for protein and coenzyme synthesis. In these organisms, sulfur is needed for sulfolipid biosynthesis, which is found in the photosynthetic membranes (Schmidt, 1988). Although surprising, the presence of strontium in Mars regolith extract was also detected in similar circumstances in other works (e.g., Olsson-Francis and Cockell, 2010), which suggests that this element is a common contaminant in MGS-1. The presence of this element is not beneficial to cyanobacteria, as it negatively affects their growth rates and chlorophyll *a* content (Foster et al., 2020). Although the composition of Mars regolith extracts allowed cyanobacterial growth in the present study, it is important to remark that extracts can be prepared under different protocols (e.g., Verseux et al., 2021), possibly allowing optimization of bioavailability of some elements and rendering it more suitable as a culture medium for defined species. In addition, there are several types of Mars regolith with different compositions that can perform better for cyanobacteria/microalgae growth (^2^ assessed on 21 February 2022).

Besides the main task of producing oxygen, a claimed application for cyanobacteria is utilization as biofertilization agents (Chittora et al., 2020). Diazotrophic cyanobacteria (e.g., *Anabaena* sp. and *Nostoc* sp.) are particularly pointed out as eco-friendly biofertilizers due to their ability to fix atmospheric nitrogen in specialized structures and deliver it to plants (Rashid et al., 2016). Furthermore, cyanobacteria improve soil porosity and water holding capacity, produce adhesive substances and excrete phytohormones, vitamins, and amino acids (Chittora et al., 2020). Indeed, their successful application as biofertilization agents was already reported for several vegetable cultures (e.g., barley, oat, tomato, radish, cotton, sugarcane, maize, chili, and lettuce) (Chittora et al., 2020). The nutrients necessary to grow plants are present is Mars (Verseux et al., 2016), and several crop cultures (e.g., tomato, rye, carrot, and garden cress) were already proven to grow on Mars regolith, developing at similar rates on Martian and low-nutrient Earth soil (Wamelink et al., 2014). Although these are exciting results, there are few organic molecules on Mars (e.g., Heinz and Schulze-Makuch, 2020) that can support overall soil fertility in the long term, and plants cannot fix atmospheric nitrogen, which indicates that the enrichment with nitrogen, like that operated by diazotrophic cyanobacteria, is very important (Verseux et al., 2016). Arai et al. (2008) demonstrated that a terrestrial *Nostoc* sp. HK-01 can successfully grow in Mars regolith, releasing polysaccharides that contributed to converting regolith into organic soil. The ability of *A. cylindrica* to release K, Mg, Na, Ca, Fe, Mn, Ni, Sr, Cu, Li, and Zn from a basalt analog was also shown (Olsson-Francis and Cockell, 2010), and this feature can increase the odds for successful plant growth on Martian regolith. Also, the use of the lysate of diazotrophic cyanobacteria previously grown on Mars and Moon regoliths for the growth of *Lemna* sp. was already reported as successful (Ramalho et al., 2020). Despite this encouraging evidence, it is fair to recognize that the culturing conditions influence the composition of cyanobacteria, mainly the carbohydrate composition (Depraetere et al., 2015). The mechanism that limits the biofertilizer role of cyanobacteria/microalgae should be considered in future research. Another constraint could be the lack of a beneficial rhizosphere, facilitating nutrient uptake by the plants. For example, for *Lemna*, several studies showed that the association with specific bacteria not only increases plant growth but also prevents oxidative stress (Ishizawa et al., 2017a,b). In the case of poor culture media, such as the Mars regolith extract, the presence of these beneficial rhizobacteria would exert a major influence.

In the present work, the duckweed *L. minor* was used as a vegetable model. This family of plants (Lemnaceae) was already pointed out as suitable for space exploration, since they are 100% edible, rich in nutrients, and fast growing (Stewart et al., 2020). Our results show that the photosynthetic efficiency, in some cases, was higher in Mars regolith extract than in the standard culture medium. This suggests that, although *L. minor* grows at a smaller pace in Mars regolith extract than in optimal medium, its healthy growth can be sustained for longer toward better overall biomass production. The treatment made to the cyanobacteria/microalgae cultures provided as biofertilization agents (filtration or sonication) did not affect the photosynthetic efficiency of *L. minor*. In addition, it is worth noting that the photosynthetic efficiency of *L. minor* did not differ among treatments with cyanobacteria and sole Mars regolith extract culturing, while treatments where the microalga was supplied as a biofertilizer harmed the photosynthetic efficiency of the plant. Actually, despite the fact that *C. vulgaris* is one of the most studied species for space life-support systems, some of its by-products were already proven to be toxic to higher plant crops (Terskov et al., 1979). However, while the number of *Lemna* fronds increased more throughout the experiment in the control with the synthetic medium than in any of the other treatments, the dry weight yield recorded for plants growing in Mars regolith extract supplemented with *S. platensis* and *C. vulgaris* is similar to that recorded in the control. These are the two species that grew worst in Mars regolith extract, which indicates that even low biomass levels may have interesting biofertilization potential. Analysis of the composition of *Lemna* growth media showed relevant levels of potassium, nitrates, and orthophosphates (major constraints of plant growth; Goopy and Murray, 2003) in *A. platensis* samples, which concur to explain why these were the treatments better promoting *Lemna* growth. *C. vulgaris* samples were not particularly rich in any of these elements, which suggests that the presence of other unmonitored compounds is responsible for their biofertilization potential. Indeed, previous studies, such as the work of Refaay et al. (2021), already showed the positive contribution of *A. platensis* and *C. vulgaris* in the culturing of several vegetable species due to their high levels of proteins, carbohydrates, and important phytohormones, such as abscisic, gibberellic, and indoleacetic acid.

In spite of a higher duplication rate (reflected in frond numbers), the plant culture in the synthetic medium did not produce higher biomass (reflected in dry weight) than the cultures in Mars regolith supplemented with these cyanobacteria and the microalga, and certain questions arise regarding the differential nutritional value of the cultured plants. Indeed, Chakrabarti et al. (2018) showed that the nutritional profile depends on the cultivation conditions. Duckweeds have an attractive nutritional profile, bearing high protein levels and essential amino acids in levels within the range recommended by WHO for good human nutrition, beneficial fatty acids (Appenroth et al., 2017), and antioxidant carotenoids (Stewart et al., 2020). A preliminary analysis of our samples by FTIR-ATR separated the nutritional profile (polysaccharides in particular) of the plants grown in regolith and supplemented regolith from those grown in synthetic media and ultrapure water. Plants are important sources of carbohydrates, which are responsible for providing energy to humans. These carbohydrates can be divided into energy storage carbohydrates, like starch, oligosaccharides, and sugars, and structural cell wall polysaccharides (Lovegrove et al., 2017), which differ in features such as their digestibility. Given the importance of these features when considering crop production on Mars to support colonization attempts, differences in the nutritional profiles of different experimental treatments, such as the ones noticed herein, are a critical object for future research. For instance, an in-depth analysis of major nutritional groups (i.e., proteins, carbohydrates, lipids, minerals, and fibers) is required to gain a robust insight into the nutritional value of vegetable species grown in Martian soil. A final note is worth making on the capacity of duckweeds to accumulate protective carotenoids, such as zeaxanthin, which can play an important role in providing protection from high levels of damaging radiations (Stewart et al., 2020). Therefore, future research should also address the production of these compounds.

## Data Availability Statement

The original contributions presented in this study are included in the article/Supplementary Material, further inquiries can be directed to the corresponding author/s.

## Author Contributions

IM contributed to the conceptualization, methodology, data treatment, and writing. TV did the conceptualization, performed the methodology, and carried out the data treatment. SF did the conceptualization and performed the methodology. JS wrote, reviewed, and edited the manuscript. HP and CS performed the methodology. FG supervised the data and wrote, reviewed, and edited the manuscript. SV and JP did the conceptualization, wrote, reviewed, and edited the manuscript, and supervised the data. All authors contributed to the article and approved the submitted version.

## Conflict of Interest

The authors declare that the research was conducted in the absence of any commercial or financial relationships that could be construed as a potential conflict of interest.

## Publisher’s Note

All claims expressed in this article are solely those of the authors and do not necessarily represent those of their affiliated organizations, or those of the publisher, the editors and the reviewers. Any product that may be evaluated in this article, or claim that may be made by its manufacturer, is not guaranteed or endorsed by the publisher.

## Footnotes

1
www.esa.int

2
https://sciences.ucf.edu/class/exolithlab/
