# Supplementary material for: Cyanobacteria as Candidates to Support Mars Colonization: Growth and Biofertilization Potential Using Mars Regolith as a Resource
Source: Front Microbiol. 2022 Jul 5;13:840098. doi: 10.3389/fmicb.2022.840098 (PMC9295076; doi:10.3389/fmicb.2022.840098)
Supplement: Supplementary file 1 [file Data_Sheet_1.PDF]

Supplementary Material

Table S1. MGS-1 Mars Global Simulant bulk chemistry, according to the manufacturer (Exolith Lab).

**Compound**	**Weight percent measured by TXRF**
SiO_2	45.6
TiO_2	0.3
Al_2O_3	9.4
Cr_2O_3	0.1
FeO_T	16.9
MnO	0.1
MgO	16.5
CaO	4.0
Na_2O	3.7
K_2O	0.4
P_2O_5	0.4
SO_3	2.6

Table S2: ANOVA summary table for the elements analyzed in the media used for *L. minor* growth test (Mars regolith extract + cyanobacteria/microalgae). Test (*F*) value and corresponding P-value, as well as corresponding degrees of freedom and mean squares of the error (MSresidual) are shown.

Source of variance	df	MSresidual	F	P
Sulphur	8, 9	7699.621	30.637	< 0.001
Calcium	8, 9	390.495	22.681	< 0.001
Potassium	8, 9	0.641	88.484	< 0.001
Manganese	8, 9	0.000101	21.742	< 0.001
Nitrates	1, 2	0.170	0.0588	0.831
Orthophosphate	7, 8	0.0294	153.490	< 0.001

.

Species	Day 7 (MGS-1, H_2O, Synthetic medium)	Day 14 (H_2O, MGS-1, Synthetic medium)
Nostoc muscorum	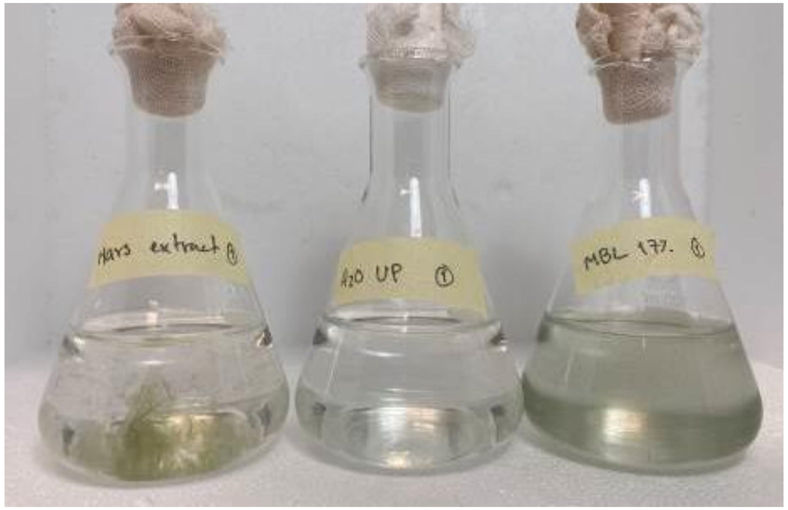	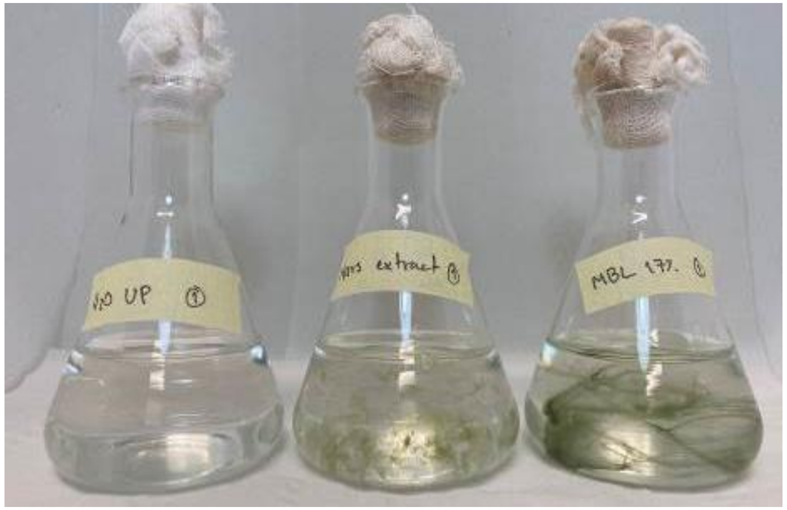
	**Day 21** (MGS-1, H_2O, Synthetic medium)	**Day 25** (MGS-1, H_2O, Synthetic medium)
	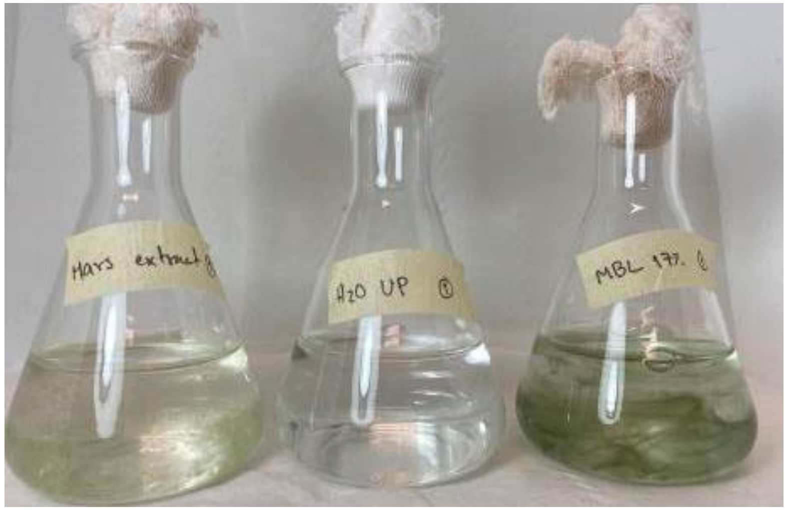	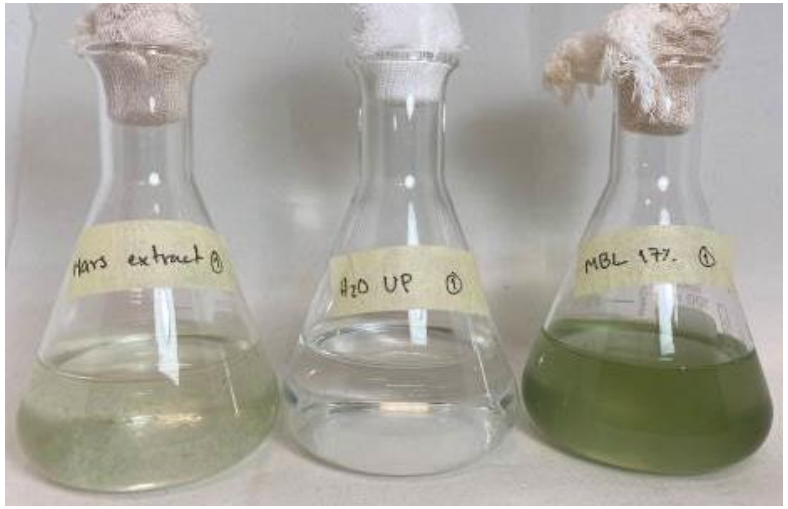

Species	Day 7 (H$_2$O, MGS-1, Synthetic medium)	Day 14 (H$_2$O, MGS-1, Synthetic medium)
Anabaena cylindrica	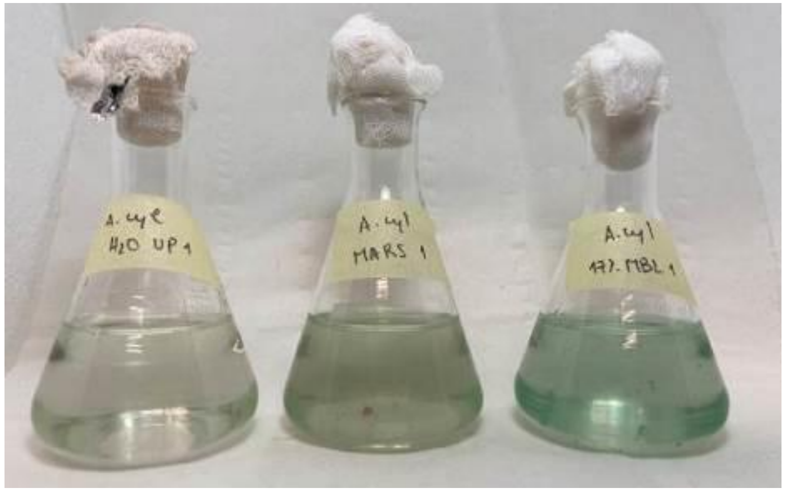	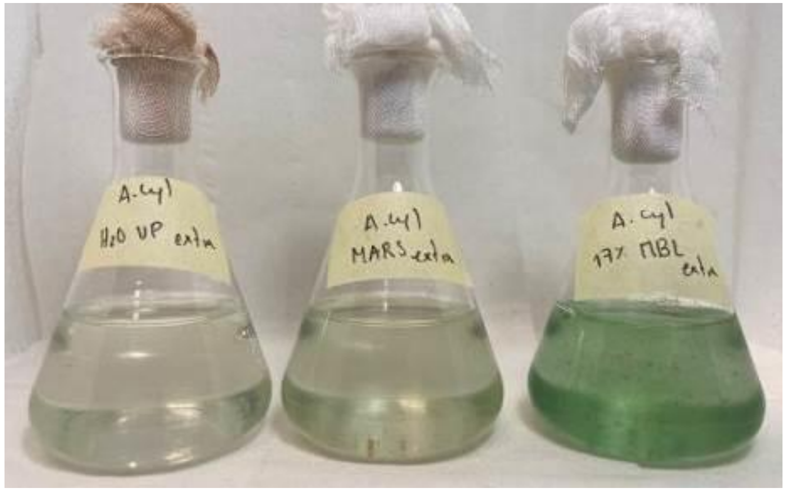
	**Day 21** (H$_2$O, MGS-1, Synthetic medium)	**Day 25** (H$_2$O, MGS-1, Synthetic medium)
	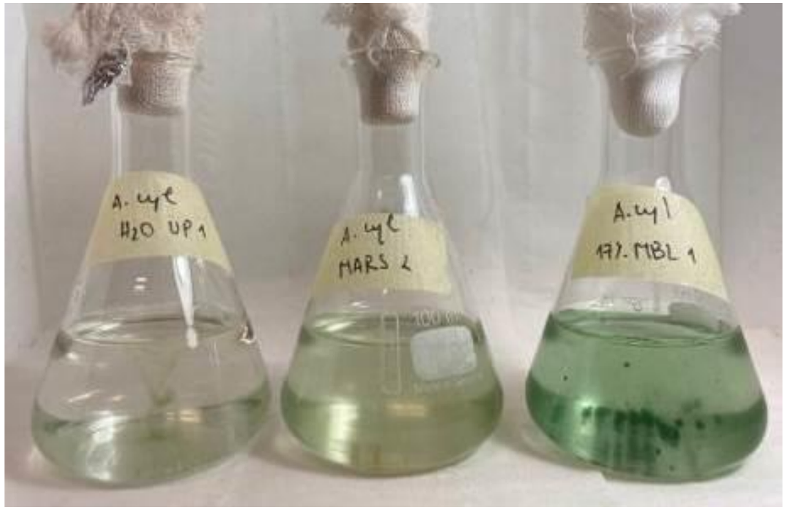	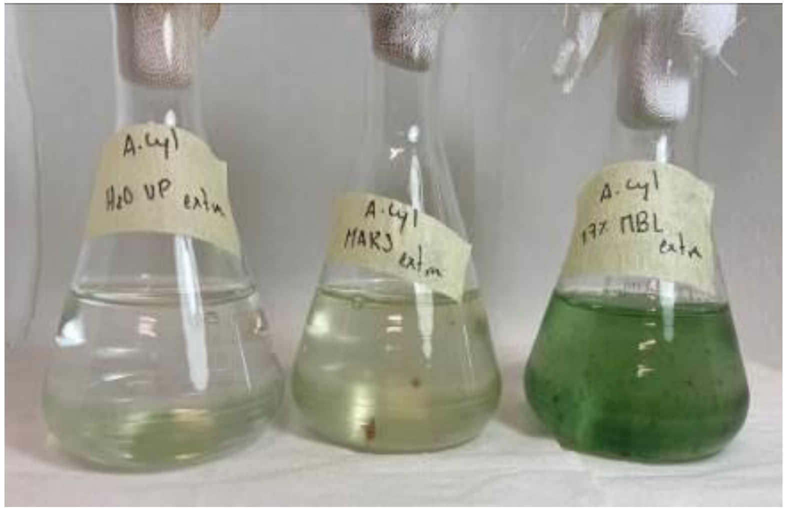

frontiers

Species	Day 7 (H$_2$O, MGS-1, Synthetic medium)	Day 14 (H$_2$O, MGS-1, Synthetic medium)
Arthrospira platensis	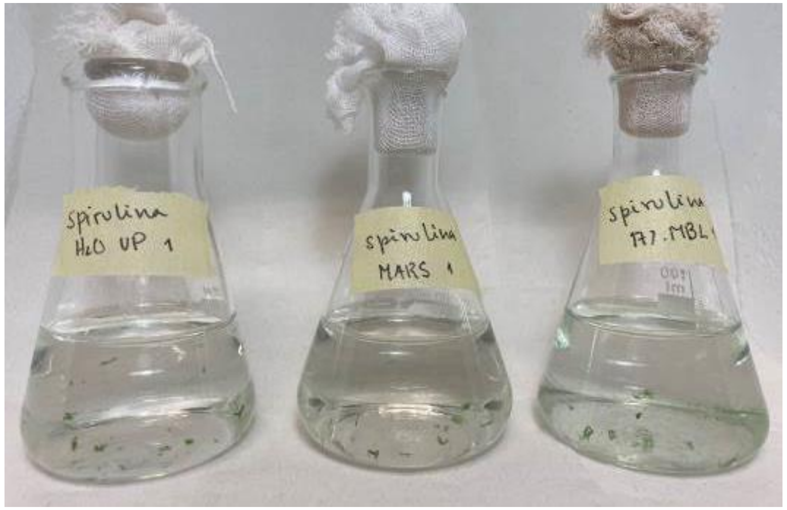	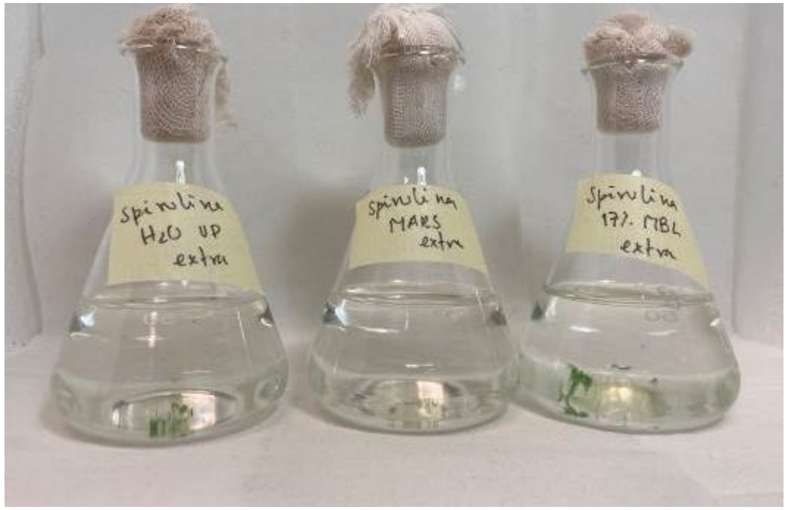
	**Day 21** (H$_2$O, MGS-1, Synthetic medium)	**Day 25** (H$_2$O, MGS-1, Synthetic medium)
	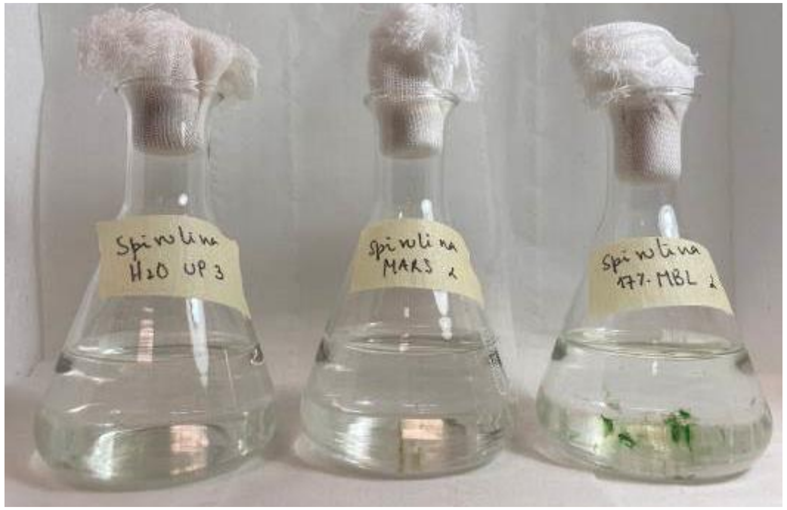	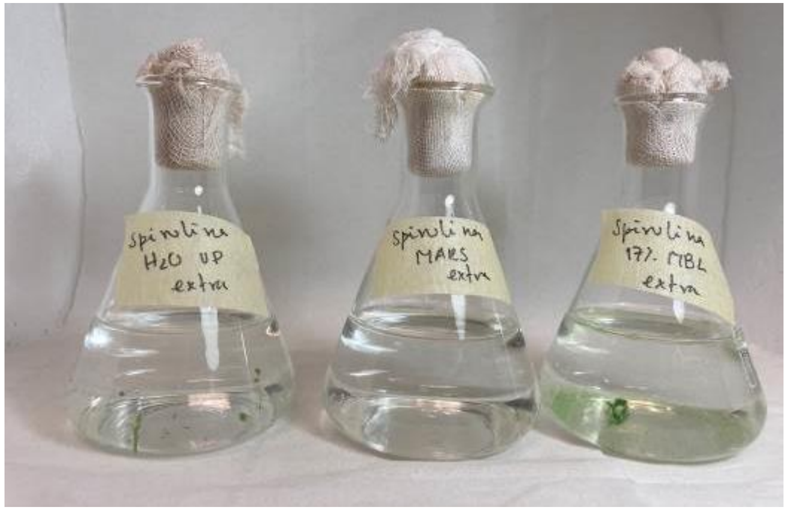

Species

Chlorella vulgaris

Day 7 **(H_2O, MGS-1, Synthetic medium)**

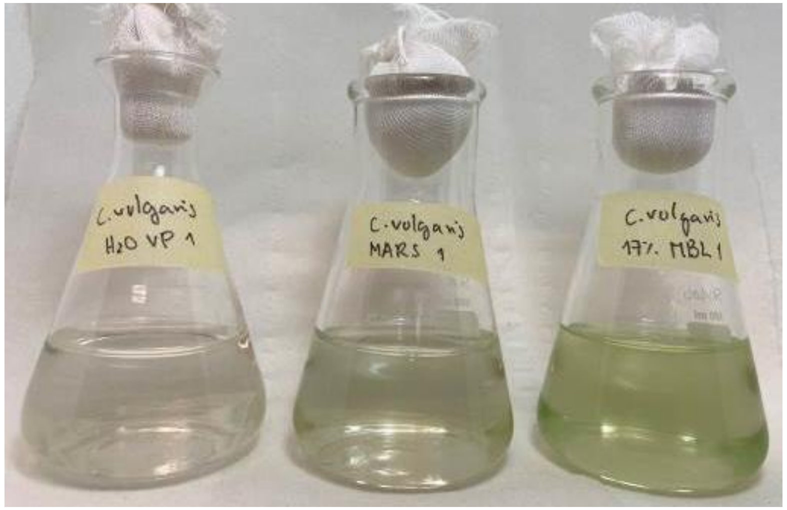

Day 14 **(H_2O, MGS-1, Synthetic medium)**

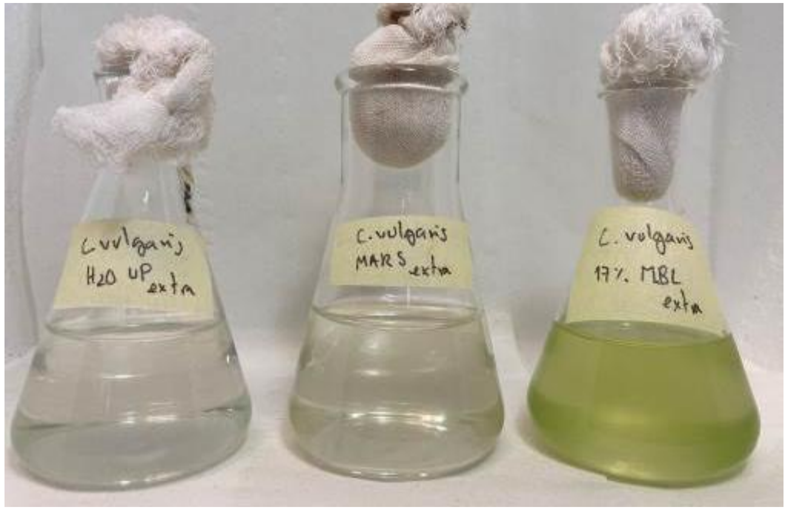

Day 21 **(H_2O, MGS-1, Synthetic medium)**

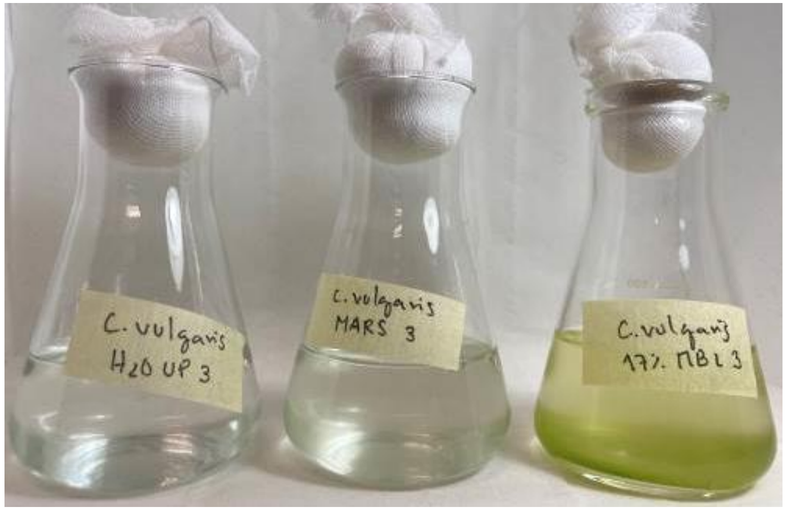

Day 25 **(H_2O, MGS-1, Synthetic medium)**

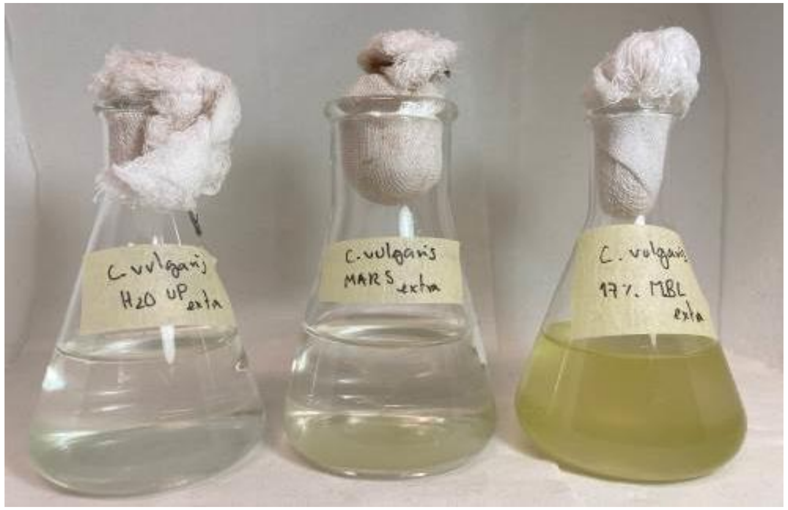

Figure S1: Pictures of the cyanobacteria/microalgae cultures growing during the experiment. Treatments are specified above each picture

Treatments			
Mars regolith extract	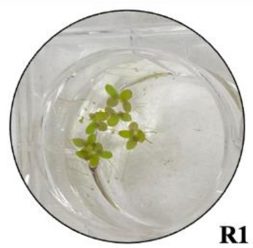 R1	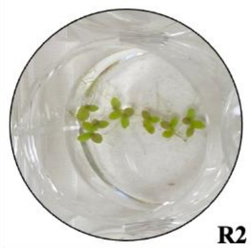 R2	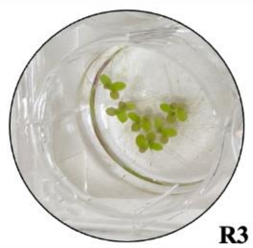 R3
Steinberg medium	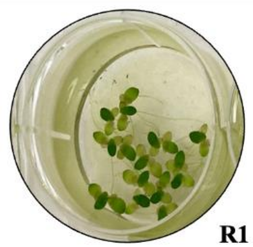 R1	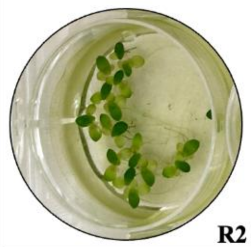 R2	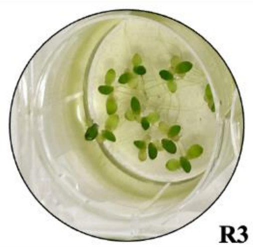 R3
Ultrapure water	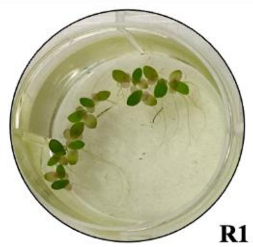 R1	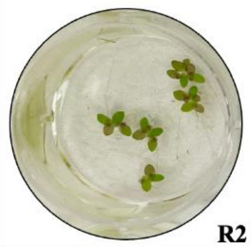 R2	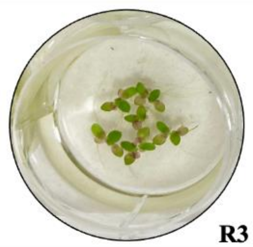 R3

Nostoc muscorum

Filtered extract

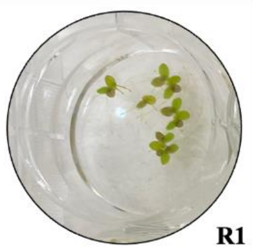

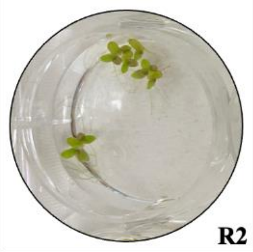

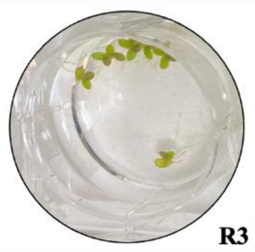

Sonicated extract

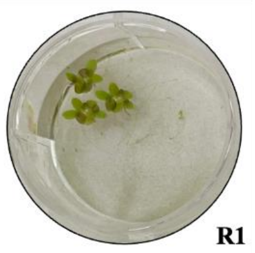

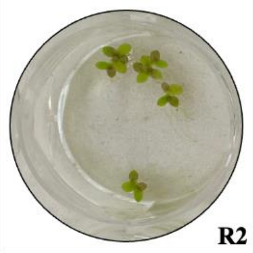

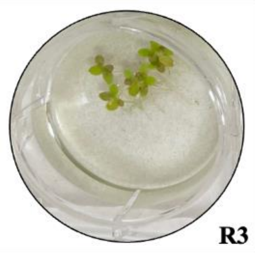

Anabaena cylindrica

Filtered extract

Sonicated extract

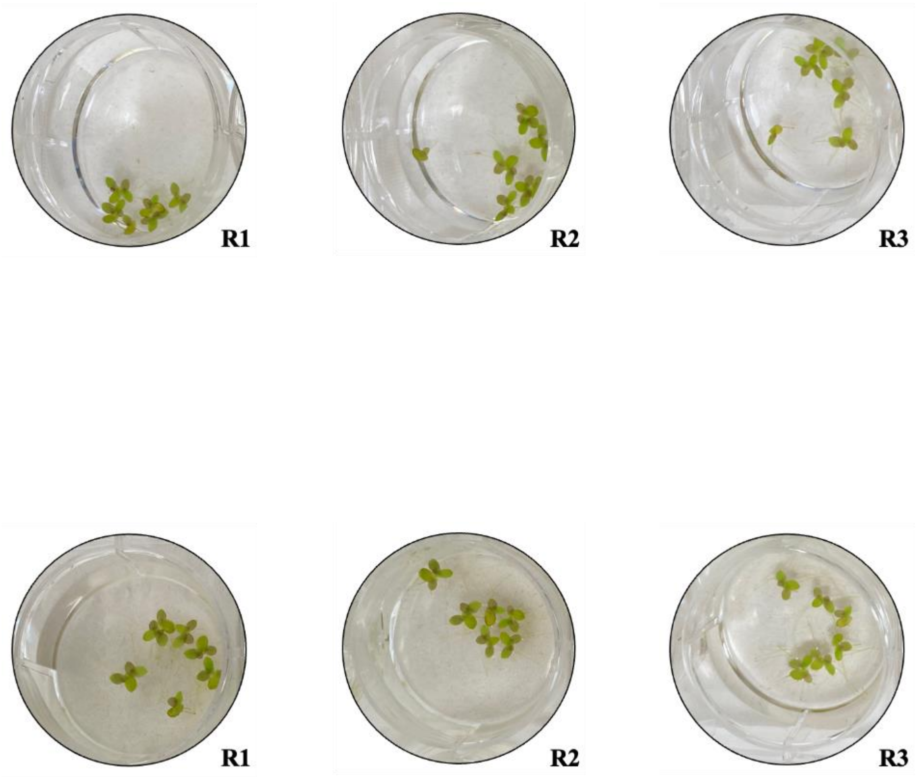

Arthrospira platensis

Filtered extract

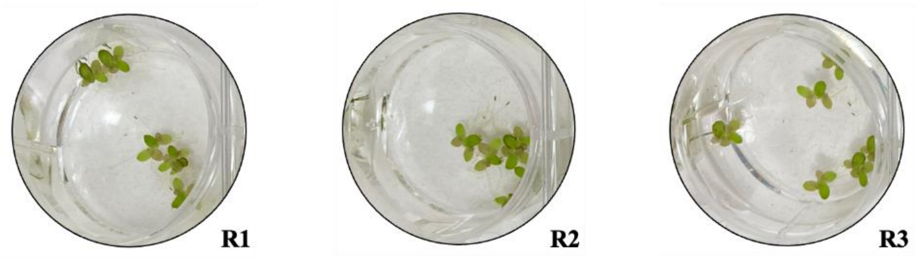

Sonicated extract

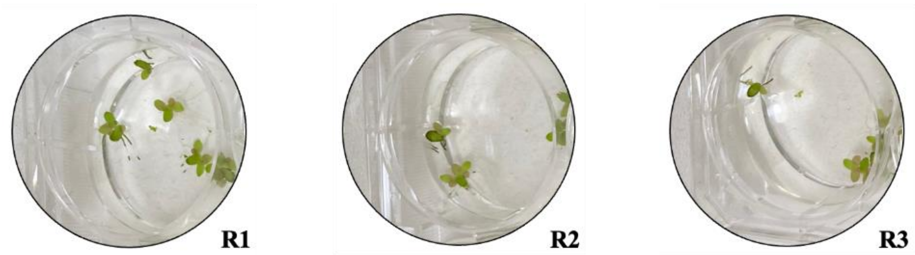

Chlorella vulgaris	Filtered extract	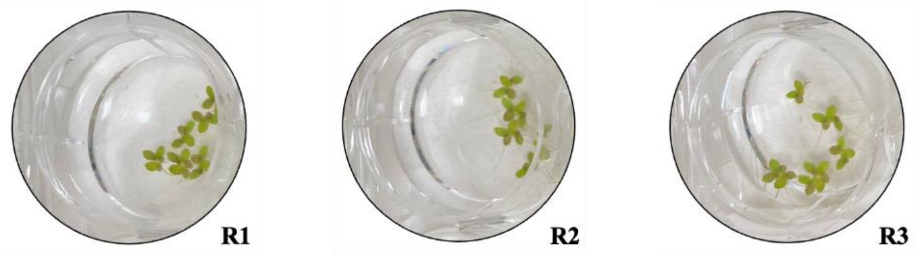
	Sonicated extract	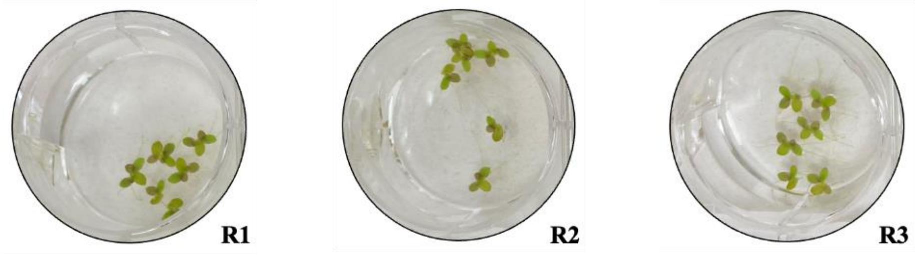

Figure S2: Pictures of the day 7 of the *Lemna minor* growth experiment.

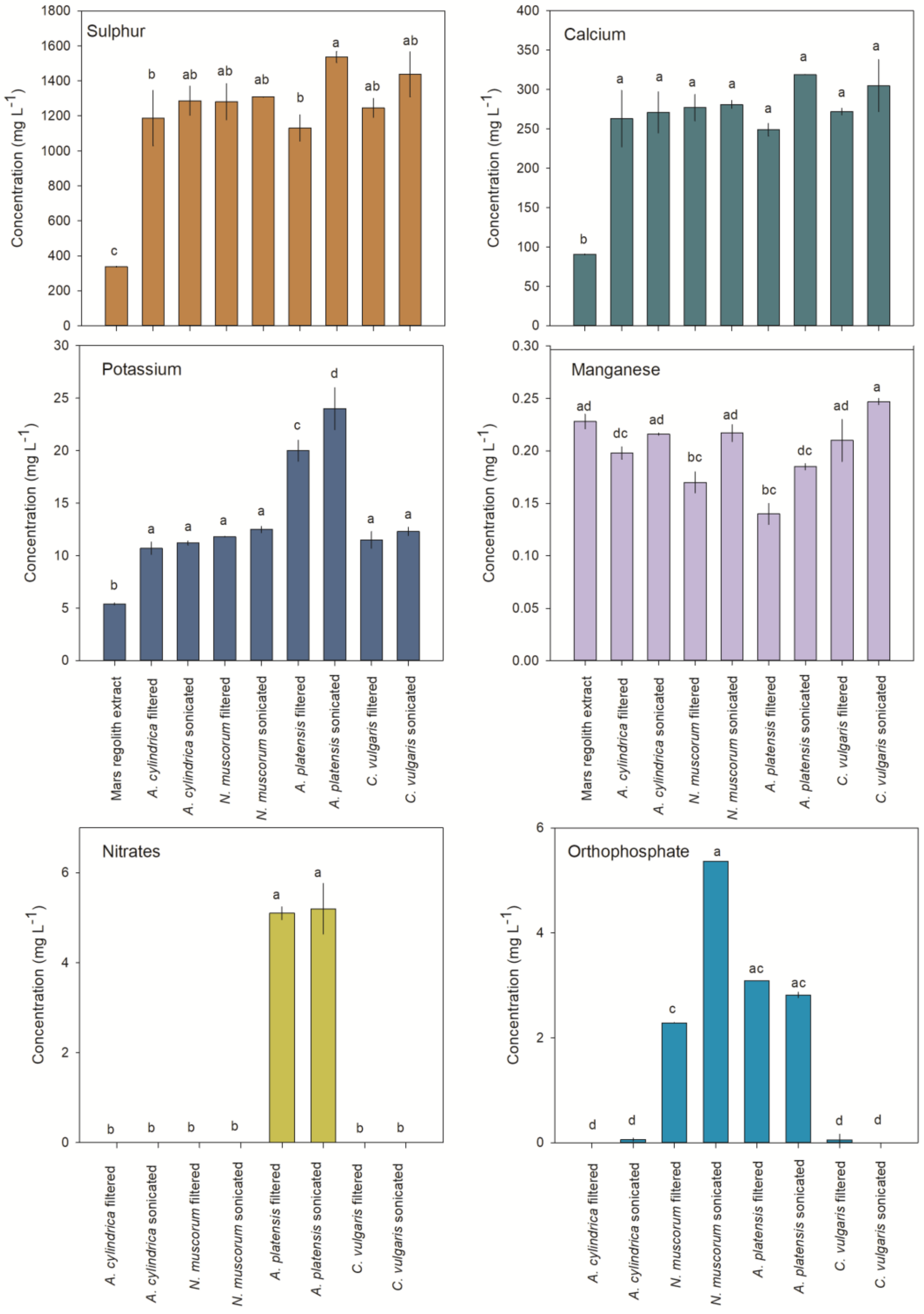

Figure S3: Analysis on the composition of the media used for *L. minor* growth test (Mars regolith extract + cyanobacteria/microalgae). Bars represent the mean of two replicates and the error bars represent the standard deviation. Different letters indicate significant differences among treatments as retrieved trough the Tukey test ($p < 0.05$) preceded by one-way ANOVA.
